# Preeclampsia Incidence and Its Maternal and Neonatal Outcomes With Associated Risk Factors

**DOI:** 10.7759/cureus.31143

**Published:** 2022-11-06

**Authors:** Bisma Khan, Razia Allah Yar, Ayesha khan Khakwani, Sajilah Karim, Hafiz Arslan Ali

**Affiliations:** 1 Department of Zoology, University of Central Punjab, Lahore, PAK; 2 Obstetrics and Gynecology, Nishtar Hospital, Multan, PAK

**Keywords:** maternal and fetal outcomes, proteinuria, hypertension, preeclampsia, fetal morbidity, arrhythmia

## Abstract

Background and objective

Preeclampsia is a hypertensive disorder that usually arises after 20 weeks of pregnancy. It is considered a major cause of maternal and fetal mortality worldwide. High blood pressure and high proteinuria are the two main characteristics of preeclamptic patients. Preeclampsia leads to either severe or mild conditions, but in both cases, it affects the organs of the mother and fetus. This study was conducted to determine the prevalence of preeclampsia and associated risk factors (family history, age, hypertension, and diabetes) and to investigate its fetal and maternal outcomes.

Methodology

This prospective study was conducted at three healthcare units in the Multan district and involved patients with gestational hypertension. Patients were diagnosed on the basis of blood pressure values, urine tests, and through Doppler ultrasound. Further investigations were conducted, including a complete hemogram and a 24-hour test for proteinuria. Results for preeclampsia-related maternal and perinatal outcomes were documented and statistical analysis was performed to analyze the data.

Results

A total of 142 patients were diagnosed with gestational hypertension and preeclampsia during the two-year study period. Our findings showed 8.67% cases of gestational hypertension and 3% of preeclampsia. The majority of the preeclamptic patients were less than 24 years of age (33.3%), belonged to lower socioeconomic classes (44.4%), and had low educational levels (81.1%). A close association of family history (36.67%) with diabetes (15.5%) and chronic hypertension (5.55%) was observed in these patients. Maternal and fetal outcomes were related to maternal blood pressure. A significant incidence of premature births (45.6%) and a majority of cesarean cases (63.4%) with severe complications were observed. Data from preeclamptic patients showed high albuminuria levels (42.2%) with problems like renal infection, pulmonary edema, and severe anemia. During the study period, a neonatal death rate of 11.1% was observed as well as issues like respiratory tract syndrome, asphyxia, and growth retardation.

Conclusion

This study showed that poor economic and educational levels are significantly associated with this disease. A high rate of maternal and neonatal morbidity with neonatal mortality was investigated. Mild to severe outcomes were observed in the form of cesarean deliveries and preterm births. Serious complications lead to ICU admissions causing a serious burden on healthcare units. Paying more attention to the healthcare needs of pregnant women helps to identify preeclampsia earlier and also minimizes the complications associated with it.

## Introduction

Preeclampsia is a major medical condition associated with maternal and fetal mortality and morbidity. Preeclampsia is spreading worldwide, especially in underdeveloped and developing countries including Pakistan [1]. It is a pregnancy-associated disease that affects the heart and other body organs such as kidneys and lungs, and further damages liver functions in severe cases. This condition is linked to the complexities of hypertension and proteinuria (albuminuria). Preeclampsia usually begins after 20 weeks of pregnancy [2].

An expecting woman has high blood pressure during preeclampsia, which is higher than or equal to 140/90 mmHg of systolic and diastolic blood pressure, as well as albumin protein in the urine. The albumin that is normally present in the blood during kidney damage is released into the urine. More than 30 mg/L of albumin indicates kidney damage and the presence of these two crucial complications at the same time in pregnancy may point to preeclampsia [3].

Preeclampsia entails either severe or mild manifestations. Mild symptoms include pain in the chest, vomiting, high blood pressure, low amount of urine, and obesity whereas severe pain in the abdomen, low breathing, kidney failure, edema, blurred vision, and eye irritation are the symptoms of severe preeclampsia, which can evolve to eclampsia in some cases. Eclampsia is a harmful stage in which premature birth and, occasionally, fetal death occur. Blood pressure is not controlled by medication in eclampsia in some cases, leading to mental abnormalities and for this reason, this complication is also referred to as the last stage of preeclampsia [4].

Preeclampsia is associated with several sociodemographic and environmental factors, which often accelerate the development of the disease, such as a family history of hypertension, heart disease, kidney disease, and diabetes [5]. Regular consumption of an unhealthy diet before and during pregnancy also leads to preeclampsia. It is important to avoid using alcoholic products in diet [6]. A diabetic woman is at a higher risk of pregnancy-associated preeclampsia as compared to non-diabetic pregnant women. Diabetes that already exists before pregnancy (preexisting diabetes) is associated with a more complicated pregnancy and higher severity in preeclamptic patients [7].

Hypertension is the major factor based on which preeclampsia is diagnosed, and it is controlled by antihypertensive medications in pregnancy. Specialists also recommend magnesium sulfate and corticosteroid medicines to reduce the rate of eclampsia and associated complications that in turn lead to rectifying fetus health and help with the growth of the baby [3]. Obesity (abnormal weight gain) is a factor that is not only linked to preeclampsia but also a major cause of cardiac diseases. Obesity in pregnant women can also have a negative impact on fetal outcomes (premature birth, abnormal growth) on top of maternal outcomes such as diabetes, heart disease, and hypertension. Maternal age, multiple pregnancies, kidney dysfunction, and in vitro fertilization are some other factors that increase the risk of preeclampsia [8].

Preeclamptic incidents are on the rise worldwide, and the condition's prevalence is greater in developing countries as compared to the developed world [9]. According to the World Health Organization (WHO), the incidence of preeclampsia ranges between 2% and 10% of pregnancies worldwide. About 1.8-16.7% of the incidents are reported in developing countries, while in developed countries, the rate is 0.4% [10]. Pakistan is a developing country and it accounts for high levels of preeclamptic incidents (as high as 5%) in pregnant women [11].

## Materials and methods

The present study was conducted at Seyal Medical Centre, Asghari Tariq Hospital, and Nishtar Hospital in the Multan district in the Punjab province of Pakistan for two consecutive years (2020-2022). The study focused on patients with more than 20 weeks of gestation. To investigate the rate of preeclampsia, risk factors, and neonatal and maternal outcomes, all patients visiting the Obstetrics and Gynecology department during the study period were included in the study, while for studying gestational hypertension, a separate, shorter study was conducted during this time period with a limited number of patients. The research work involved diagnosis, data collection, and case study observations. The selected patients were admitted to the Department of Obstetrics and Gynecology of the referred healthcare units. Admitted patients were consulted directly and through their attendants to collect data on their sociodemographic variables such as age, family history, economic status, educational level, physical activity, any type of social support, and obstetric history including signs and symptoms, number of pregnancies, and other complications. Blood pressure, urine test, and Doppler ultrasounds were performed at local centers in the presence of specialists and technicians for the purpose of disease diagnosis. Further testing was performed, including a complete hemogram, coagulation profile, renal and liver function tests, and a 24-hour proteinuria test. The same protocol was followed for obstetric management at all healthcare units. The details of labor pertaining to whether it was induced or spontaneous along with the mode of delivery were recorded. Maternal complications before and after delivery were observed in detail along with neonatal complications. Patients with severe cases, multiple pregnancies, cardiac disease, kidney failure, and neurological disorders were excluded from the study. Ethical approval (Individual Consent Letter with reference number ICL-111 ) from concerned departments was obtained as well as informed oral consent from the participants to share their data for research purposes. At the end of the study, the results were compiled and analyzed.

## Results

A total of 52 out of 600 patients were observed to have hypertension during pregnancy; of these, 32.69% were preeclampsia cases. A total of 90 preeclampsia cases were observed among 2,800 patients visiting the Obstetrics and Gynecology departments of the concerned hospitals. Family history, chronic hypertension, gestational diabetes, and patient age were analyzed as associated risk factors for hypertensive patients (Tables 1-2).

**Table 1:** Association between hypertensive disorders and diabetes among pregnant women (n=52)

Variable		N		%
Gestational hypertension		21		40.38
Chronic hypertension		9		17.3
Preeclampsia		17		32.69
Eclampsia		5		9.6
Gestational diabetes				
Yes	19		36.53	
No	33		63.46	

**Table 2:** Sociodemographic characteristics of patients with hypertension (n=52)

Factors	N	%
Age (years)		
<20	6	11.5
20-30	28	53.8
31-40	13	25
>40	5	9.61
Educational status		
Primary	18	34.6
Secondary	25	48.07
Above secondary	9	17.3
Family history		
Yes	14	26.92
No	38	73.07

The data for hypertensive patients were further analyzed and categorized based on elevated blood pressure values and the presence of albumin protein in the urine (Table 3).

**Table 3:** Hypertensive clinical features among pregnant women (n=52)

Factors		N	%
Patient’s blood pressure at the time of diagnosis (mmHg)			
≤140/90		19	36.54
>140/90-160/110		27	51.9
≥160/110		5	9.6
Patient’s pulse at the time of diagnosis (bpm)			
≤86		7	13.46
>86-90		13	25
91-98	23		44.23
>98	9		17.3
Proteinuria			
Yes	22		40.38
No	30		59.6

For maternal and fetal outcomes, a total of 90 patients with preeclampsia were observed in detail. These patients were further investigated for sociodemographic characteristics, clinical features, and maternal and neonatal outcomes. The predominant age group was ≤24 years, and more than half of the subjects (62.2%) were from urban areas. A high percentage of middle- (n=42, 46.75%) and low-income (n=40, 44.45%) patients were observed to have preeclampsia as compared to patients of high economic status (n=8, 8.89%). In this study, 33 (36.67%) women had a family history of preeclampsia. Severe preeclampsia was diagnosed in 20 (22.23%) patients, and 32 (35.56%) had mild stage while 38 (42.22%) subjects were at the normal stage of preeclampsia (Tables 4-5).

**Table 4:** Sociodemographic characteristics of participants with preeclampsia (n=90)

Factors	N	%
Age (years)		
≤24	30	33.3
25-29	26	28.9
30-34	14	15.5
≥35	20	22.2
Residence		
Rural	34	37.8
Urban	56	62.2
Economic status		
High	8	8.89
Middle	42	46.7
Low	40	44.4
Family history of preeclampsia		
Yes	33	36.67
No	57	63.33
Stages of disease		
Normal	38	42.22
Mild	32	35.56
Severe	20	22.23

**Table 5:** Demographic characteristics of preeclamptic patients (n=90)

Variable	N	%
Patient’s educational status		
Primary	29	32.2
Secondary	44	48.9
Above secondary	17	18.9
Patient’s occupational status		
Housewife	47	52.3
Private teacher	16	17.8
Government employee	6	6.67
Laborer	12	13.3
Saleswoman	4	4.4
Nurse	2	2.2
Banker	3	3.33
Husband’s educational status		
Primary	37	41.11
Secondary	32	35.6
Above secondary	21	23.4
Husband’s occupational status		
Laborer	29	32.3
Farmer	8	8.9
Private job	34	37.8
Government job	15	16.7
Businessman	4	4.4

Most of the respondents (n=44, 48.9%) had received at least a secondary level of education (excluding those with only primary levels). A high number of the patients (n=47, 52.3%) were housewives whereas the life partners of a high number of patients (n=34, 37.8) had private jobs; among the life partners of patients, 41.11% (n=37) had only a primary-level educational (Table 5) while 32 had received a secondary-level education.

In this study, 14 (15.5%) of the preeclamptic subjects were diabetic, 10 (11.1%) were diagnosed with anemia, and obesity was observed in six (6.7%) women. Eight (8.9%) patients had hypocalcemic-complicated pregnancies, four (4.5%) were asthmatic, and three (3.33%) subjects had arrhythmia. Among the remaining patients, five (5.55%) had hypertension at the time of diagnosis. Two women (2.22%) had a hernia, and nine (10.0%) had multiple complications (preeclampsia with diabetes and obesity, preeclampsia with anemia and hypocalcemia, preeclampsia with diabetes, hernia, and anemia) whereas 28 (31.1%) had preeclampsia without other comorbidities (Table 6).

**Table 6:** Prevalence of multiple disorders among preeclamptic women (n=90)

Variable	N		%
Preeclamptic only	28		31.1
Diabetes	14		15.5
Anemia	10		11.1
Obesity	6		6.7
Hypocalcemia		8	8.9
Arrhythmia		3	3.33
Asthma		4	4.5
Thrombocytopenia		1	1.11
Hypertension		5	5.55
Hernia		2	2.22
Multiple comorbidities		9	10.0

The predominant clinical features among the participants were elevated blood pressure, pulse, and proteinuria levels. Forty-six (51.1%) participants had systolic/diastolic blood pressure of less than or equal to 140/90 mmHg whereas 38 (42.3%) women had a blood pressure of more than 140/90 mmHg; there were six (6.7%) severely hypertensive patients. About 30 (33.3%) subjects had abnormal pulse rates ranging from 91 to 98; 22 patients (24.5%) had a history of abdominal surgery, with higher chances of premature birth. Seven (7.8%) women had a history of abortion, while 13 (14.5%) women had a history of kidney disease, which is a risk factor for preeclampsia. The majority of the patients (n=52, 57.8%) had more than 0.3-3 g/L protein levels in the urine based on 24-hour urine testing (Table 7).

**Table 7:** Clinical features related to preeclampsia among pregnant women (n=90)

Variables	N	%
Patient’s blood pressure at the time of diagnosis (mmHg)		
≤140/90	46	51.1
>140/90-160/110	38	42.3
≥160/110	6	6.7
Patient’s pulse at the time of diagnosis (bpm)		
≤86	23	25.6
>86-90	20	22.3
91-98	30	33.3
>98	17	18.9
Gestational diabetes		
Yes	35	38.9
No	55	61.1
Abdominal surgery history		
Yes	22	24.5
No	68	75.6
Abortion history		
Yes	7	7.8
No	83	92.2
Kidney disease history		
Yes	13	14.5
No	77	85.6
Proteinuria (g/24h)		
>0.3-3 g/L	52	57.8
>3 g/L	38	42.2

No maternal mortality was observed in this study. Maternal outcomes like mode of delivery and clinical complications like pulmonary and renal diseases leading to admissions to ICU were reported. It was observed that most of the women (n=57, 63.4%) had cesarean deliveries as compared to vaginal (n=33, 36.7%). Fifty-six (62.2%) patients fully recovered without any severe outcomes after delivery; 17 (18.9%) patients had low Hb levels and seven (7.8) had kidney infections. Pulmonary edema was reported in six (6.6%) patients. Four (4.4%) patients faced serious conditions and were hence admitted to ICU for closer observation (Table 8).

In the present study, it was observed that 41 (45.6%) births were premature, and 19 (21.1%) of these preterm babies were delivered between 34 and <37 weeks. Six (6.67%) of the babies had extremely premature births. There were 10 (11.1%) neonatal deaths, and four (4.45%) of these deaths were due to low birth weight. Among the total 90 births, 46 (51.1%) had normal birth weights, ranging from 2.5 kg to 4 kg. Among the 80 babies who survived, 32 (35.6%) had breathing problems, eight (8.89%) were growth-retarded, and 12 (13.3%) were mentally abnormal (Table 9).

**Table 8:** Maternal outcomes among the study respondents (n=90)

Outcomes	N	%
Mode of delivery		
Cesarean	57	63.4
Vaginal	33	36.7
Maternal death	0	0.0
Complications		
Kidney infection	7	7.8
Admission in ICU	4	4.4
Pulmonary edema	6	6.6
Low Hb levels	17	18.9
Fully recovered	56	62.2

**Table 9:** Fetal outcomes of preeclampsia among pregnant women (n=90)

Variables	N	%
Premature birth		
Yes	41	45.6
No	49	54.5
Premature birth (weeks)		
≤28	6	14.63
28-33	16	39.02
34-<37	19	46.34
Neonatal death		
Yes	10	11.1
No	80	88.8
Birth weight (kg)		
1-1.5	24	26.7
1.6-2.4	20	22.2
2.5-4	46	51.1
Fetal death complications		
Low birth weight	4	4.45
Pneumonia	2	2.22
Stillbirth	1	1.11
Respiratory tract syndrome	3	3.33
Alive fetal complications		
Growth retardation	8	8.89
Asphyxia	5	5.56
Hypothermia	7	7.78
Breathing problem	32	35.6
Mental retardation	12	13.3
Low RBCs	16	17.78

Data comparison revealed that elevated blood pressure increased the chances of cesarean deliveries and premature birth rates. High blood pressure had a great impact on the mode of delivery: all six (6.6%) patients with ≥160 mmHg systolic and 110 mmHg diastolic blood pressure had cesarean deliveries; 27 (30%) patients with greater than 140/90 mmHg to 160/110 mmHg blood pressure had cesareans. However, 22 (24.5%) of women who had vaginal deliveries had ≤140 mmHg systolic and 90 mmHg diastolic values (Table 10).

**Table 10:** Association between mode of delivery and maternal blood pressure levels (n=90)

Variables	N	%	N	%
Patient’s blood pressure at the time of diagnosis (mmHg)	Cesarean	Cesarean	Vaginal	Vaginal
≤140/90 (n=46)	24	52.17	22	47.82
>140/90-160/110 (n=38)	27	71.05	11	28.94
≥160/110 (n=6)	6	100	0	0.0

High blood pressure levels also had an effect on premature birth rates. A total of 41 premature births were documented in this study. Among these, 12 (13.3%) of newborns had preterm births due to the mother’s blood pressure being ≤140/90 mmHg, and mothers of 23 (25.6%) premature babies had a mild blood pressure of >140/90 mmHg to 160/90 mmHg. However, all women with extremely high blood pressure levels (≤160/90 mmHg) had premature babies (n=6, 6.7%) (Table 11).

**Table 11:** Association of premature birth rate (n=41) with maternal blood pressure levels

Patient’s blood pressure at the time of diagnosis (mmHg)	Total (n=90)	Premature birth (n=41)	%
≤140/90	46	12	26.08
>140/90-160/110	38	23	60.52
≥160/110	6	6	100

## Discussion

This two-year study was conducted at three healthcare units in the Multan district. This study focused on finding incidences and complications linked with preeclampsia and their effect on maternal and fetal mortality rates. Due to hypertensive disorders during pregnancy, there are high incidences of maternal and fetal mortality in Pakistan. In developing countries, the estimated rate of preeclampsia is 1.8-16.7%. In the current study, it was much lower than the peak value (3%) and also much lower than that found in another study conducted in Pakistan, in the Sukkur district, where it was 5% [12]. A study conducted in Dhaka, Bangladesh showed a 14% incidence with less incidence in the rural areas (26.1%) compared to urban areas (73%), which slightly contrasts with the present study. The present study revealed a 37.8% incidence in rural areas and 62.2% in urban areas [10]. In a study conducted in Ethiopia, 12.4% of the patients were preeclamptic. In contrast to the present study, they included only those cases who were older than 35 years (more likely to have preeclampsia because of lack of a good diet, awareness, and low economic status). However, in the present study, the predominant age group was ≤24 years, which is similar to the findings by Mou et al. (2021) [10] and also comparable with the studies conducted by Soomro et al. (2019) [12], Belay and Wudad [13], Wassie and Anmut [14], Ugwu et al. (2011) [15], and Singhal et al. (2009) [16] (Table 12).

**Table 12:** A comparison of sociodemographic characteristics of preeclamptic women among various studies

Factors	Present study	(Soomro et al., 2019) [12]	(Mou et al., 2021) [10]	(Ugwu et al., 2011) [15]
Preeclampsia percentage	3%	5%	14.4%	3.3%
Rural residence	37.8%	Rural study	26.1%	------
Urban residence	62.2%	-------	73%	Urban study
Age group	≤24 years (33.3%)	20-29 years (48.7%)	<25 years (36.9%)	20-29 years (50.6%)
Educational level	Secondary (48.9%)	Uneducated (60.3%)	Secondary (37.7%)	Secondary (46.7%)
Occupation (housewife)	52.3%	------	96.3%	------
Maternal mortality	0%	------	------	0%
Neonatal mortality	11.1%	------	------	15%

Educational level, occupation, and economic standards affect the rise of preeclampsia cases [16-17]. A study held at the Gandhi Memorial Hospital in Ethiopia concluded that preeclampsia was associated with sociodemographic characteristics such as gestational weeks, age, and economic status [14]. In the present study, 52.3% of patients were housewives by occupation, and this was lower (%) than the finding in the study at the El-Shatby Maternity University Hospital in Alexandria where 57.8% of women were housewives [18]. A much higher figure was observed by Mou et al. in 2021 [10]. Family history is another major factor that increases the risk of preeclampsia. In the current study, 36.67% of women had a family history of preeclampsia. A study conducted based on the National Health Insurance Database of Taiwan in 2021 found that 12.17% of the cases had a family history of preeclampsia, and these women were also at great risk for hypertension [5]. The obstetrical characteristics (diabetes mellitus) of the respondents were similar to the study conducted by Belay and Wudad [13].

It has been observed that maternal and fetal outcomes are linked to risk factors associated with preeclampsia. Elevated systolic and diastolic values also affect premature birth and mode of delivery. Severe neonatal and maternal complications are related to the onset and severity of disease including zero to varying numbers of both maternal and neonatal death rates as reported in various studies. A study conducted in 2022 by Wassie and Anmut on eclampsia outcomes reported three maternal deaths [14]. This result was almost comparable to that in the study conducted in Enugu, Nigeria, which reported zero maternal deaths [15] and less comparable with those studies where 8% and 10% of maternal deaths were reported [13-16]. In contrast with these two studies but similar to the Nigerian study, we found no maternal deaths in our study, which may be attributed to the fact that the disease was diagnosed on time and quick treatment was initiated in our cohort.

During the study period, various maternal complications were reported, including renal infection, pneumonia, and respiratory problems, with a zero death rate, which is comparable to the study performed in western Kenya. In the present study, cesarean cases were higher (approximately 63.4%) due to complications during pregnancy induced by preeclampsia, while vaginal deliveries amounted to 36.7%, which is comparable to the above-mentioned study, which also reported a high proportion of pregnancies (more than two-thirds) resulting in cesarean sections. These authors also reported 9.4% of fetal deaths associated with severe complications, which is similar to our findings; we noted that 11.1% of neonatal deaths were due to respiratory tract syndrome, low birth weight, and other related complications [17]. In a study conducted at the El-Shatby Maternity University Hospital in Alexandria, it was concluded that 4.4% of the babies had low birth weights, and this aligns with the findings of the present study in which 4.45% of the babies had low birth weights [18].

The current study has some limitations. Primarily, our findings cannot be generalized to the whole population as this was a hospital-based study with a limited number of patients. Two different cohorts of populations were used for assessing preeclampsia and hypertensive disorders incidence with a much lower number for the latter one, which was conducted as a supplementary work. Moreover, the present study’s observational design could not provide deeper insights as to which type, how much, and to what extent an educational intervention would help in knowledge improvement regarding preeclampsia or reduce adverse clinical outcomes. Furthermore, more research work in the current field is required to estimate the potential maternal-neonatal benefits of improved dietary intake and home-monitoring interventions for pre- and post-delivery preeclampsia management.

For data collection, two different questionnaires were designed for the two conditions (hypertension and preeclampsia) by reviewing past work with related research objectives. The questionnaires were investigator/administration-approved and used for data collection from enrolled participants. The information collected included patient history, sociodemographic characteristics, and clinical characteristics with maternal and neonatal complication/outcome details (see Appendices).

## Conclusions

In the current study, the incidence of preeclampsia was relatively low. Based on our findings, family history, maternal age, economic status, low education level, severe anemia, chronic hypertension, and gestational diabetes were the major factors associated with disease onset and severity. A high number of cesarean cases were reported in this study, and 41 (45.6%) deliveries were premature. During the study period, no maternal death was observed, although there was a fetal mortality rate of 11.1% as well as major complications like low birth weight, growth retardation, and respiratory tract syndrome. Maternal and fetal outcomes were highly related to elevated blood pressure levels.

## Appendices

Preeclampsia study sample proforma

Concerned hospital: …………………………………………………………………………..

Date:  -----------------------                                                             Proforma serial no: …………              

Patient’s oral consent:

(Oral permission for ethical purposes)

Statement:

Before the beginning of the study, an informed oral consent should be taken from the women after explaining the aim of the study and its phases. The participants should be assured of the confidentiality of their personal information.

Personal Details:                                                                                           Clinical features

                                                                                                                    Date of 1st visit:

1. District/city/Teh.:  …………………………                                                    BP:                     Pulse

2. Residency:                                                                                          2nd visit:                                                                       Rural/Urban                                                                      BP:                     Pulse

3. Age:       …………………..                                                                         3rd visit:

4. Pregnancy week: …………………………….                                                BP:                     Pulse

5. Education Level: 

                       Primary/Middle/secondary school level/Above

6. Occupation:

                        Housewife/Private Job/Govt. Job      

7. Husband education level:

                        Primary/Middle/secondary school level/Above

8. Husband Job:

                          Labour/Private Job/Govt. Job      

9. Per month family earning:  (value in Rs)

10. Economic level:

                              High (> 50K) _________

                               Middle (20 to 50K) _______

                                Low (< 20K) _________

11. Pregnancy:

                     1st/2nd/3rd/other

12. Siblings no:     

13. Diabetes:             Yes/No                      Type:…………………………………………

14. Chronic hypertension:     Yes    No

15. Any cardiac disease:         Yes    No  

    One          multiple          (mention all)

16. Any surgery in past:          Yes    No

                                                 Abdominal           cardiac             other

17. Any other disease:

18. Proteinuria report:  add value

Maternal and neonatal outcomes

19. Type of delivery:

                                Normal               cesarean               preterm               full term

20. In case of preterm’ gestational week:   ……………………………

21. Maternal complication/s after delivery:………………………………………………

22. Neonatal complication/s:……………………………………………………………

Fetal birth weight (gm):

23. NICU admission:                   Yes           No   (specify)

24. NICU admission reason: 1.…………………………………………………………………………………………………………………………………………………………........  2.……………………………………………………………………………………………………………………………………………………………….

25. Neonatal death:                     Yes          No

Reason………………………………………………………………………………………………………………………………………………………………………………………………………………………………………………………………………………………………………………................................

26. Maternal death:                    Yes          No

Reason………………………………………………………………………………………………………………………………………………………………………………………………………………………………………………………………………………………………………………

27. Any other significant activity:
 

Hypertension and preeclampsia (data collection proforma)

Concerned hospital:…………………………………………………………………………..

Date:  -----------------------                                                             Proforma serial no:…………              

Patient’s oral consent:

(Oral permission for ethical purposes)

Statement:

Before the beginning of the study, an informed oral consent should be taken from the women after explaining the aim of the study. The participants should be assured of the confidentiality of their personal information.

Personal Details:                                                                                           Clinical features

                                                                                                                    Date of 1st visit:

1. District/city/Teh.:  …………………………                                                       BP:

2. Residency:                                                                                               2nd visit:                                                                    Rural/Urban                                                                   BP:

3. Age:       …………………..                                                                             3rd visit:

4. Pregnancy week: …………………………….                                                   BP:

5. Education Level: 

                       Primary/Middle/secondary school level/Above

6. Occupation:

                        Housewife/Private Job/Govt. Job      

7. Husband education level:

                        Primary/Middle/secondary school level/Above

8. Husband Job:

                          Labour/Private Job/Govt. Job      

9. Economic level:

                           Low/middle/high

10. Per month family earning:     (value in Rs)

11. Pregnancy:

                     1st/2nd/3rd/other

12. Siblings no:     

13. Diabetes:             Yes/No                      Type:…………………………………………

14. Chronic hypertension:     Yes    No

15. Any cardiac disease:         Yes    No   

16. Any surgery in past:          Yes    No

17. Any other disease:

18. Proteinuria report:  add value

19. Any other significant activity:

## References

[1] Rana S, Lemoine E, Granger JP, Karumanchi SA (2019). Preeclampsia: pathophysiology, challenges, and perspectives. Circ Res.

[2] Filipek A, Jurewicz E (2018). Preeclampsia - a disease of pregnant women (Article in Polish). Postepy Biochem.

[3] Askie LM, Duley L, Henderson-Smart DJ, Stewart LA (2007). Antiplatelet agents for prevention of pre-eclampsia: a meta-analysis of individual patient data. Lancet.

[4] Ramos JG, Sass N, Costa SH (2017). Preeclampsia. Rev Bras Ginecol Obstet.

[5] Wu CT, Kuo CF, Lin CP, Huang YT, Chen SW, Wu HM, Chu PH (2021). Association of family history with incidence and gestational hypertension outcomes of preeclampsia. Int J Cardiol Hypertens.

[6] Staff AC (2019). The two-stage placental model of preeclampsia: an update. J Reprod Immunol.

[7] Weissgerber TL, Mudd LM (2015). Preeclampsia and diabetes. Curr Diab Rep.

[8] Stubert J, Reister F, Hartmann S, Janni W (2018). The risks associated with obesity in pregnancy. Dtsch Arztebl Int.

[9] Osungbade KO, Ige OK (2011). Public health perspectives of preeclampsia in developing countries: implication for health system strengthening. J Pregnancy.

[10] Mou AD, Barman Z, Hasan M, Miah R, Hafsa JM, Das Trisha A, Ali N (2021). Prevalence of preeclampsia and the associated risk factors among pregnant women in Bangladesh. Sci Rep.

[11] Yeo S (2010). Prenatal stretching exercise and autonomic responses: preliminary data and a model for reducing preeclampsia. J Nurs Scholarsh.

[12] Soomro S, Kumar R, Lakhan H, Shaukat F (2019). Risk factors for pre-eclampsia and eclampsia disorders in tertiary care center in Sukkur, Pakistan. Cureus.

[13] Belay AS, Wudad T (2019). Prevalence and associated factors of pre-eclampsia among pregnant women attending anti-natal care at Mettu Karl Referral Hospital, Ethiopia: cross-sectional study. Clin Hypertens.

[14] Wassie AY, Anmut W (2021). Prevalence of eclampsia and its maternal-fetal outcomes at Gandhi Memorial Hospital, Addis Ababa Ethiopia, 2019: retrospective study. Int J Womens Health.

[15] Ugwu EO, Dim CC, Okonkwo CD, Nwankwo TO (2011). Maternal and perinatal outcome of severe pre-eclampsia in Enugu, Nigeria after introduction of magnesium sulfate. Niger J Clin Pract.

[16] Singhal SR, Deepika P, Anshu S, Nanda S (2009). Maternal and perinatal outcome in severe pre-eclampsia and eclampsia. JSAFOG.

[17] Irene K, Amubuomombe PP, Mogeni R, Andrew C, Mwangi A, Omenge OE (2021). Maternal and perinatal outcomes in women with eclampsia by mode of delivery at Riley mother baby hospital: a longitudinal case-series study. BMC Pregnancy Childbirth.

[18] Yakout SM (2016). Impact of physical stretching exercise on feto-maternal outcomes among mild preeclamptic pregnant women in Egypt. Am J Nurs Sci.

